# Characterization of Photo-Cross-Linked Polyethylene Pipes for Geothermal Energy Storage

**DOI:** 10.1021/acsomega.4c09896

**Published:** 2025-01-03

**Authors:** Giulia Simão de Sousa, Andrew Root, Ivo Heinmaa, Adib Kalantar Mehrjerdi, José Roberto Moraes d’Almeida, Mikael Skrifvars

**Affiliations:** † Swedish Centre for Resource Recovery, Faculty of Textiles, Engineering and Business, University of Borås, 501 90 Boras, Sweden; ‡ Department of Chemical and Materials Engineering, Pontifical Catholic University of Rio de Janeiro, 22451-900 Rio de Janeiro, Brazil; § MagSol, 00970 Helsinki, Finland; ∥ National Institute of Chemical Physics and Biophysics, 12618 Tallinn, Estonia; ⊥ MuoviTech AB, 507 30 Brämhult, Sweden

## Abstract

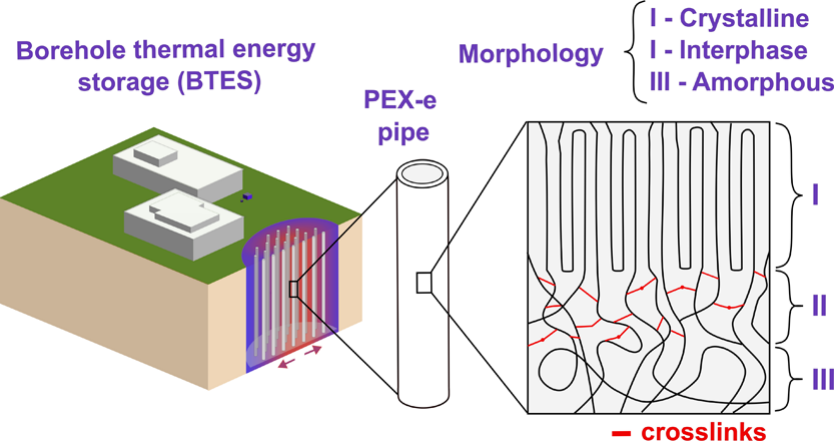

This study investigates the morphology and thermo-mechanical properties of cross-linked polyethylene (PEX) pipes for potential use in high-temperature borehole thermal energy storage systems. Particular attention is given to a novel type of PEX pipe produced through photoinitiated cross-linking (PEX-e). Two formulations, PEX-e1 and PEX-e2, were analyzed and compared to peroxide-cross-linked polyethylene (PEX-a) and non-cross-linked bimodal polyethylene (PE100) pipes. The degree of cross-linking was evaluated via gel content, while cross-link density and molecular weight between cross-links were determined using dynamic mechanical analysis (DMA). Phase composition and molecular mobility were explored through ^1^H static nuclear magnetic resonance (NMR), and the melting and crystallization behavior was assessed by differential scanning calorimetry (DSC). Oxidative stability and degradation were examined by using Fourier transform infrared (FTIR) spectroscopy, oxidation induction time (OIT) measurements, and thermogravimetric analysis (TGA). Both PEX-e formulations achieved satisfactory cross-linking degrees and exhibited remarkable OIT values. However, significant differences in cross-link distribution were noted, with PEX-e2 showing a less uniform dispersion of cross-links, which resulted in a lower storage modulus. FTIR analysis indicated that oxidation products were formed in PEX-e1 during cross-linking, highlighting the need for further optimization of the formulation and processing conditions.

## Introduction

In response to the ongoing climate and energy crisis, thermal energy storage (TES) systems have emerged as a promising solution for extending the time gap between energy production and energy consumption. Among the various approaches available, borehole thermal energy storage (BTES) is often preferred due to its relatively low initial costs and minimal environmental impact.^1^ In BTES systems, a network of borehole heat exchangers transfers heat to and from the surrounding rock or sediment mass, allowing heat to be stored during the summer for use in winter space heating, or vice versa.^1−3^ High-temperature BTES systems have also been used to store waste heat from shopping malls, data centers, and industrial facilities, repurposing it for district heating.^2,4^

Borehole heat exchangers (BHE) consist of heat exchange pipes through which a heat transfer fluid is circulated via pumping.^2,3^ BHE pipes are typically made from high-density polyethylene (HDPE) and cross-linked polyethylene (PEX), with the latter being often preferred for high-temperature applications due to its superior thermal resistance.^2,5^

Cross-linking is a well-established method to improve the durability and thermal resistance of PE pipes.^6−9^ Several methodologies have been developed for cross-linking PE, typically involving free-radical reactions initiated by chemical agents or physical processes. Among these, the photo-cross-linking process has recently gained significant commercial interest for pipe manufacturing^10,11^ and is being explored as a potential material for high-temperature BTES systems.

Currently, PEX-a is the most widely used type of PEX for potable water transport and heating applications.^12,13^ This process involves the use of a peroxide cross-linking agent, such as di-*tert*-butyl cumyl peroxide (BCUP) or dicumyl peroxide (DCP).^14^ At temperatures above the melting point of polyethylene, the peroxide decomposes and abstracts hydrogen from the polymer chain, creating polyethylene radicals that form covalent bonds (cross-links) with adjacent radicals.^15,16^ However, this process has several drawbacks, including a low cross-linking rate, heat sensitivity, and the production of unwanted volatile byproducts, which require extended degassing at high temperatures.^17^ As a result, PEX-e is being explored as a promising alternative due to its higher cross-linking rate (enabling greater production efficiency), lower overall energy consumption, and lack of volatile chemical emissions.^18^

In the PEX-e process, the PE formulation contains a photoinitiator additive, typically a benzophenone derivative, that absorbs UV light, undergoes photoexcitation, and can abstract hydrogen atoms from the polymer backbone.^18,19^ This reaction takes place immediately after extrusion, while the polymer remains in its melted state and therefore is transparent to visible and UV light. The addition of an auxiliary cross-linking agent (CA), such as triallylcyanurate (TAC), has been shown to significantly accelerate the cross-linking process, leading to higher degrees of cross-linking (over 70%) and a more uniform distribution of cross-links.^18−20^ In this case, three possible cross-link configurations can be formed: PE–PE, PE–CA–PE, and PE[CA]–PE, where [CA] represents the copolymer segment of the cross-linking agent. The statistical probability of each configuration depends on reaction kinetics.^18^ As a result, the cross-links in PEX-e are not solely composed of carbon–carbon bonds, as they are in PEX-a, which may influence the properties of the pipe.

Photo-cross-linking of polyethylene has been extensively studied by Rånby and co-workers.^19−26^ Several photoinitiators, cross-linking agents, and experimental conditions were explored for the photo-cross-linking of polyethylene films and continuous profiles. One key issue identified was preoxidation of the polymer during photo-cross-linking,^23^ which can negatively affect the polymer’s durability. However, it was found that optimized stabilizer formulations could significantly reduce preoxidation. Specifically, a formulation containing the antioxidant Irganox B215, along with small amounts of the hindered amine photostabilizer (Tinuvin 770), proved effective in preventing oxidation during processing.^23^

Fallani et al.^27^ investigated the mechanical properties of photo-cross-linked polyethylene films, reporting improvements in thermal stability, elastic modulus, and barrier properties. Numerous studies^18,28−32^ have also explored the reaction mechanisms of the photo-cross-linking process with PE containing different photoinitiators and coagents. Xue et al.^33^ reviewed the application of photo-cross-linking in the production of shape-memory polymers, while other studies have focused on its use in industrial cable insulation.^18,34,35^ However, there is a notable gap in the scientific literature regarding the use of photo-cross-linking for pipes and tubing.

Accordingly, the present study aims to assess the morphology and physical properties of PEX-e pipes produced by using two different formulations. For comparison, PEX-a and non-cross-linked HDPE pipes were also analyzed. The degree of cross-linking was evaluated through gel content, while cross-link density and the molecular weight between cross-links were determined using dynamic mechanical analysis (DMA). The phase composition and molecular mobility were explored via ^1^H static nuclear magnetic resonance (NMR), and the melting and crystallization properties were studied using differential scanning calorimetry (DSC). Oxidative stability and degradation were investigated through Fourier transform infrared (FTIR) spectroscopy, oxidation induction time (OIT) measurements, and thermogravimetric analysis (TGA). Finally, the dynamic mechanical response of the materials over a wide temperature range was assessed by using DMA.

## Experimental Section

### Materials

The four different pipes investigated in this study were supplied by the MuoviTech International Group, Sweden. Their characteristics are listed in Table 1. Two PEX-e pipes, namely, PEX-e1 and PEX-e2, with different formulations were investigated. According to the supplier, Formulation I (PEX-e1) contains a higher percentage of antioxidant additives compared to standard commercial formulations for drinking water, with the aim of enhancing thermo-oxidative stability for high-temperature BTES application. Since BTES systems are closed and do not allow leakage into other water sources, there is no concern about additives leaching into drinking water over time. Additionally, Formulation IV contains a 2.5% carbon black additive for UV stabilization. The supplier also indicated that PEX-e1 and PEX-e2 were manufactured under slightly different processing conditions to account for the different thicknesses in the pipes. However, specific details regarding the processing conditions were not disclosed. Further details about the additive packages are also not provided.

**Table 1:** Pipes Investigated in the Study

designation	cross-linking agents	additive package	outer diameter (mm)	thickness (mm)
PEX-e1	UV-photoinitiator + coagent	I	32	3
PEX-e2	UV-photoinitiator + coagent	II	16	2
PEX-a	peroxide	III	32	3
PE100		IV	40	4

**Figure 1:**
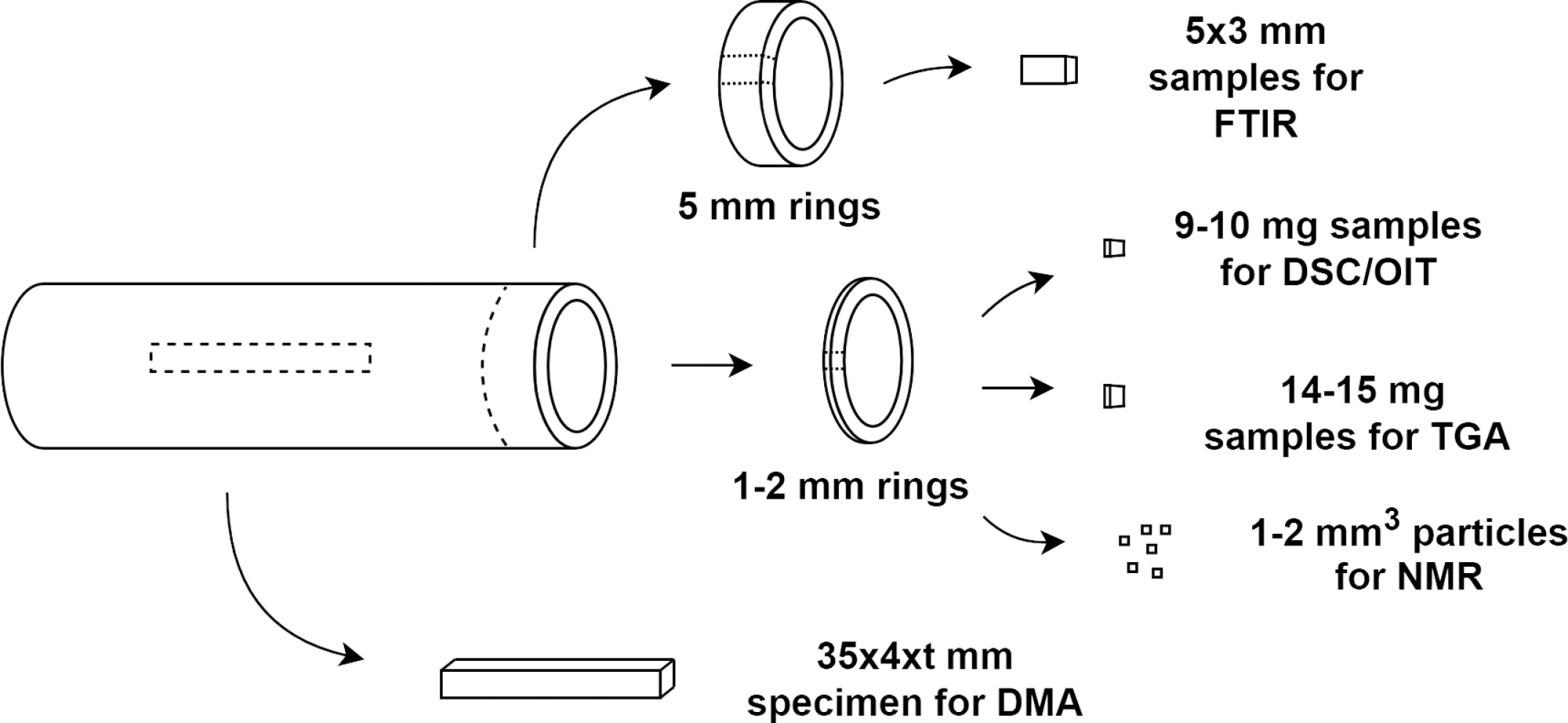
Schematics of sample preparation, where *t* stands for thickness of the pipe.

Samples for characterization were cut from the pipes according to Figure 1 in “as received” conditions. For FTIR spectroscopy, the samples were cut from the pipes by using a pipe-cutting tool. The cross-sectional rings were cut from the pipes using a microtome, and smaller sections were cut with a knife and weighted accordingly. A computer numerical control (CNC) machine was used to cut the specimens for DMA.

### Characterization Methods

#### Gel Content

The degree of cross-linking was assessed by the determination of the gel content by solvent extraction, according to the ISO 10147:2012 standard. Samples were cut from the pipes using a microtome. Slices with a thickness of (0.4 ± 0.1) mm were taken from the pipe cross-section. Two replicates of each pipe were made. The samples with mass ≥ 0.2 g were put in a cage made of stainless steel mesh with 120 μm pore size, and the gel extraction was performed in xylene solvent at 138–140 °C for 8 h. After extraction the samples were dried in a vacuum oven, kept at 90 °C at 0.85 bar for at least 3h before being weighted.

The degree of cross-linking (gel) was calculated as per eq 1:
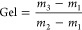
1where *m*_1_ is the mass of the cage and lid, *m*_2_ is the mass of the original test piece, the cage and the lid, and *m*_3_ is the mass of the residue, cage and lid.

#### Solid-State Proton NMR Spectroscopy

^1^H static (nonspinning sample) NMR was used to quantify the phase composition and molecular mobility of the different pipes. The experiments were carried out on a Bruker AVANCE-II spectrometer at 4.7 T magnetic field (^1^H resonance at 200 MHz) using a home-built NMR probe having a strong rf pulse (90-degree pulse = 0.9 μs) and short dead time. The NMR data were acquired using the so-called solid echo pulse sequence (90*x*-τ-90*y*-τ-acquisition).^36^ This approach is used where the NMR free induction decay (FID) signal is very short and the beginning of the decay signal can be lost in the *deadtime* of the NMR probe. The solid echo experiments were compared with the usual direct excitation experiment and the signals detected were similar.

The decay of the solid echo experiment was then decomposed into three components usually carried out for semi-crystalline polymers.^37,38^ The decay components used were an Abragam function, a Weibullian function, and an exponential function. The fitting procedure was carried out using nonlinear least-squares regression in the “peak-o-mat” software (version 1.1.9).

The Abragam component is usually associated with rigid or crystalline areas and is represented by the combination of Gaussian and sine functions, as per eq 2:^37−39^
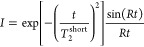
2where *I* is the normalized magnetization intensity, *R* is a fitting parameter, *t* is the decay time, and *T*_2_^short^ is the fast decay rate.

The Weibullian function is associated with the interphase region between the crystalline and amorphous regions, where motion is restricted. This function ranges between a pure Lorentzian (*n* = 1) and a pure Gaussian (*n* = 2), as per eq 3:^37−39^
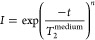
3where *T*_2_^medium^ is the intermediate decay rate. Finally, the contribution of the amorphous phase is represented by an exponential function^38^ as per eq 4:
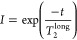
4where *T*_2_^long^ is the long decay rate. The Abragam function can be rewritten in terms of two parameters *a* and *b* as shown in eq 5:^40^
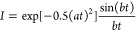
5

From the parameters *a* and *b,* the theoretical second moment can be calculated as per eq 6:^40^
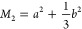
6

The second moment is a measure of the dipolar coupling between the H’s in the system and is higher the closer the H’s are to each other. If the experimental second moment is less than the expected value for a rigid lattice, it can also provide information on any motion present within that system since the value is reduced due to motional averaging.

#### Differential Scanning Calorimetry

Melting and crystallization analyses of the materials were performed on TA Q2000 DSC equipment. Samples with 9–10 mg weight were heated in aluminum pans under a nitrogen flow (50 mL/min) for two heating and one cooling cycles. First, the samples were heated from 30 to 230 °C at 10 °C/min and maintained isothermally for 3 min to erase any previous thermal history. Then, the samples were cooled down to 30 °C at 10°/min, maintained at isothermal conditions for 3 min, and heated to 230 °C at 10°/min for a second time. The degree of crystallinity (*X*_c_) was calculated according to eq 7:
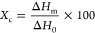
7where Δ*H*_m_ is the fusion enthalpy of the sample and Δ*H*_0_ is the equilibrium fusion enthalpy of PE crystals (281 ± 6 J/g).^41,42^ Three replicate measurements were performed for each material and the standard deviation (*S*) of *X*_c_ was calculated taking into account the error propagation, as shown in eq 8.^43^
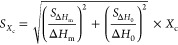
8

#### FTIR Spectroscopy

The infrared spectra were recorded in the 4000–400 cm^–1^ range with a Thermo Fisher Nicolet iS50 Spectrometer in ATR mode, with 32 scans per spectrum (4 cm^–1^). The spectra were recorded using the “OMNIC software Advanced ATR correction” which normalizes the spectrum for a penetration depth of ln(10) ≈ 2.3 μm and gives more consistent results, that are comparable to transmission mode spectra.^44^ The measurements were performed on the surface corresponding to the inside wall of the pipes. The spectra of three random samples were measured for each material to ensure reproducibility of the results.

The data preprocessing was performed separately for each absorbance spectra using Python. The baseline correction was done by asymmetric least-squares (ALS)^45^ using the *irfpy* library.^46^ The IR absorbance band at 2915 cm^–1^, characteristic of CH_2_ asymmetric stretching, was used as an internal reference to account for sample-to-sample intensity variations arising from differences in the sample thickness and curvature effects. Further information on the preprocessing and quantification of FTIR data with Python is provided as Supporting Information.

The carbonyl index has been used extensively in polymer studies as a comparative method to monitor the hydrolysis of stabilizing additives and oxidation processes. In the literature, there are different trends for calculating the carbonyl index either using normalized peak height or area.^44^ In this work, the carbonyl index was calculated according to Hiles^47^ as the integrated area of the spectral region from 1775 to 1675 cm^–1^, as per eq 9:^47^
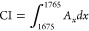
9where *A*_*x*_ is the normalized absorbance at wavenumber *x* after baseline correction. The area was calculated using numerical integration with the composite trapezoidal rule, implemented via the “trapz” function in the NumPy Python library.

#### Oxidation Induction Time

Oxidation induction time (OIT) tests were performed on TA Q2000 DSC equipment to assess the thermo-oxidative stability of the pipe samples. Samples with 9–10 mg were heated in aluminum pans to 200 °C at 20 °C/min and kept isothermal for 5 min. Then, the gas was switched from nitrogen to oxygen. The temperature was maintained at 200 °C until the maximum exothermic peak was reached. The OIT is defined as the time between the gas switching and the onset of the oxidative reaction peak.^48^ Five replicates were analyzed for each material.

#### Thermogravimetric Analysis

Thermogravimetric analysis was performed on TA Q500 TGA equipment. Samples with 14–15 mg weight were heated from 30 to 650 °C at 20 °C/min in both nitrogen and air purge stream, and the weight change as a function of temperature was recorded. The parameters obtained from the thermogravimetric (TG) and derivative (DTG) curves were *T*_5_, *T*_50_, *T*_95_, and *T*_max_. *T*_5_ is the onset temperature of decomposition, determined as the temperature at which the weight loss reaches 5%, while *T*_50_ and *T*_95_ are the temperatures at which the weight loss reaches 50 and 95%, respectively. *T*_max_ is the temperature of the maximum decomposition peak in the DTG curve.

#### Dynamical Mechanical Analysis

Dynamic mechanical thermal analysis (DMA) was performed in TA Q800 DMA equipment. PEX and PE specimens were cut from the pipes and deformed under flexure using a single cantilever arrangement at a frequency of 1 Hz. Transitions were studied in a thermal scanning mode from −50 to 170 °C at a 3 °C/min rate. Three replicates were run for each material.

The average molecular weight between cross-links (*M*_c_) was determined according to eq 10.^49,50^
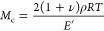
10where *E′* is the storage modulus at the rubbery plateau, ν is the Poisson ratio (ν = 0.41),^51^ ρ is the density of the amorphous polymer (ρ = 0.850 g/cm^3^),^25,52^*R* is the gas constant and *T* is the absolute temperature.

Eq 10 was derived from the statistical theory of rubber elasticity for a lightly cross-linked rubber system in very small strains. It can also be applied to semicrystalline polymers, provided that the modulus contribution from cross-links is isolated from the crystalline phase contribution.^50^ For semicrystalline polymers at temperatures above their melting point, the material becomes entirely amorphous and the storage modulus reaches a minimum or a plateau. In this plateau region, the storage modulus is a function of the molecular weight between physically effective cross-links, which includes both entanglements and chemical cross-links.^49^ For polyethylene, this plateau typically occurs around 150 to 160 °C. This was confirmed by calculating the temperature derivative of the storage modulus, which was less than 0.1 at 160 °C for all materials. Therefore the *E′* at 160 °C was used to calculate *M*_c_ for all samples. The effective cross-linking density was calculated as per eq 11.^50^
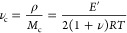
11

## Results and Discussion

### Cross-Linking Morphology

The degree of cross-linking, as determined by gel content, is summarized in Table 2. Additionally, the table includes the average molecular weight between cross-links (*M*_c_) and the cross-linking density (ν_c_), both calculated from the DMA data using eqs 10 and 11, respectively. The storage modulus at 160 °C (Table 8), where the material reaches the rubbery plateau, was used for determining the *M*_c_ and ν_c_ values. The PE100 pipe was also analyzed as a control. Since this pipe was not cross-linked, the gel content of 0.8% indicates that the experimental conditions were sufficient to dissolve the non-cross-linked portions of the PEX samples, with an experimental accuracy exceeding 99%. In general, PEX samples with higher gel content exhibited also a higher cross-linking density and a lower molecular weight between cross-links, which is in line with other studies for PEX pipes.^23,25,53,54^ In the case of PE100, the values of *M*_c_ and ν_c_ are only a measure of molecular weight between chain entanglements.

**Table 2:** Degree of Crosslinking (Gel), Molecular Weight between Crosslinks (*M*_c_), and Crosslinking Density (ν_c_) of the Pipes^a^

material	gel (%)	*M*_c_ × 10^3^ (g/mol)	ν_c_ × 10^2^ (mol/m^3^)
PEX-e1	77.1 ± 1.5	2.3 ± 0.1	3.8 ± 0.2
PEX-e2	81.0 ± 0.0	1.4 ± 0.2	6.3 ± 1.2
PEX-a	90.7 ± 0.5	0.7 ± 0.1	11.6 ± 1.4
PE100	0.8 ± 0.0	15.2 ± 0.8	0.6 ± 0.0

a Uncertainties represent the standard deviation.

The three PEX pipes exhibited different degrees of cross-linking, with PEX-a having the highest and PEX-e1 the lowest. Nevertheless, all three types met the required gel content threshold of over 70%, as specified by the standard for “plastics piping systems for hot and cold water installations” (ISO 15875-2). The gel content in PEX-e2 was only slightly higher than that in PEX-e1. However, PEX-e2 exhibited a significantly greater cross-linking density, suggesting that cross-links are less dispersed in PEX-e2. The differences between PEX-e1 and PEX-e2 may stem from variations in processing conditions^55^ and potentially from differences in additive formulations.^23^

### Molecular Dynamics and Quantitative Phase Composition

PE is a semicrystalline polymer, consisting of phases with significantly different chain mobilities.^6^ The crystalline phase typically consists of chain-folded lamellae, which, under standard processing conditions, are arranged in an orthorhombic lattice.^56,57^ In this phase, the polymer chains are highly ordered, resulting in very limited mobility. Only methyl branches and a small proportion of ethyl branches can be incorporated into the crystalline phase, while the remaining branches form an intermediate or interphase region that is more ordered than the amorphous phase.^6^ It is believed that this interphase consists of polymer chains with restricted motion compared to the amorphous phase, not only due to their proximity to the crystalline regions, but also due to chain entanglements and, in the case of PEX, cross-links.^58,59^

**Figure 2:**
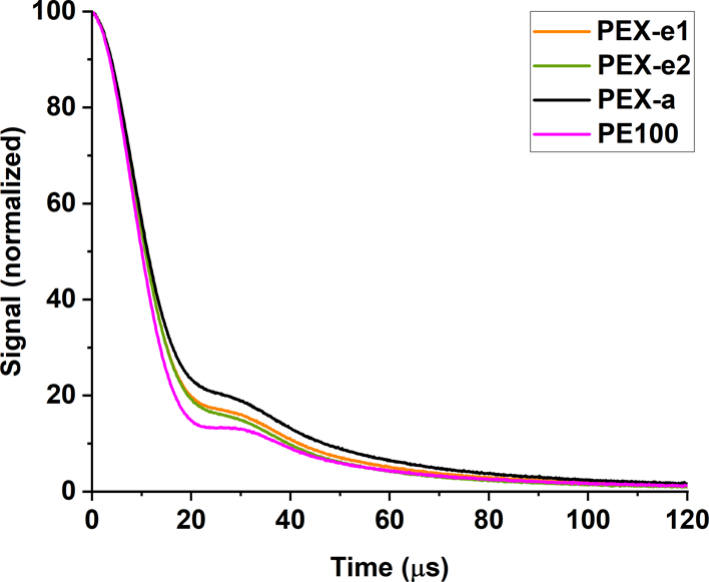
^1^H NMR solid echo decay acquired for PEX and PE pipes samples.

**Figure 3:**
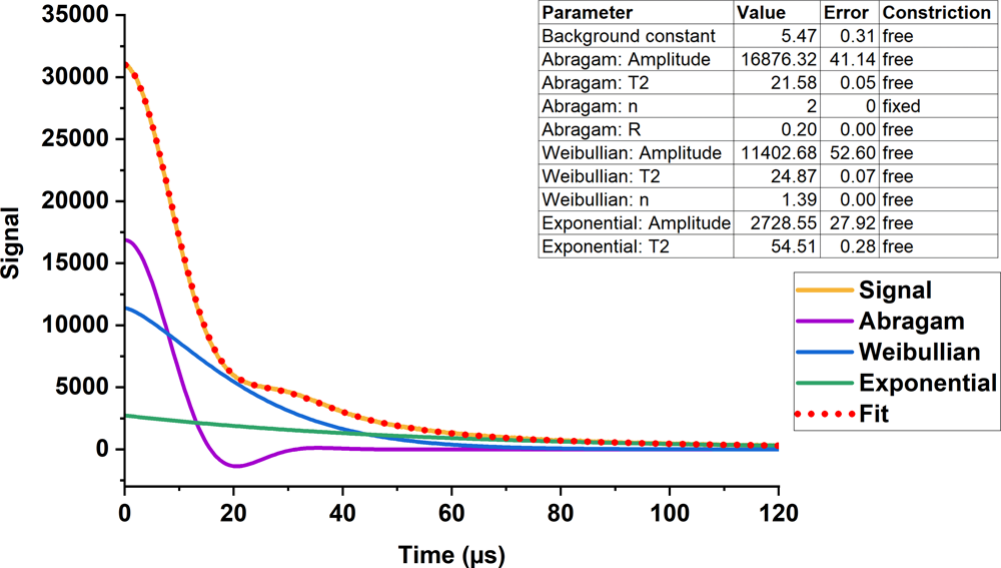
Example showing the fitting of the Abragam, Weibullian, and exponential functions to the ^1^H NMR signal of PEX-e2.

**Figure 4:**
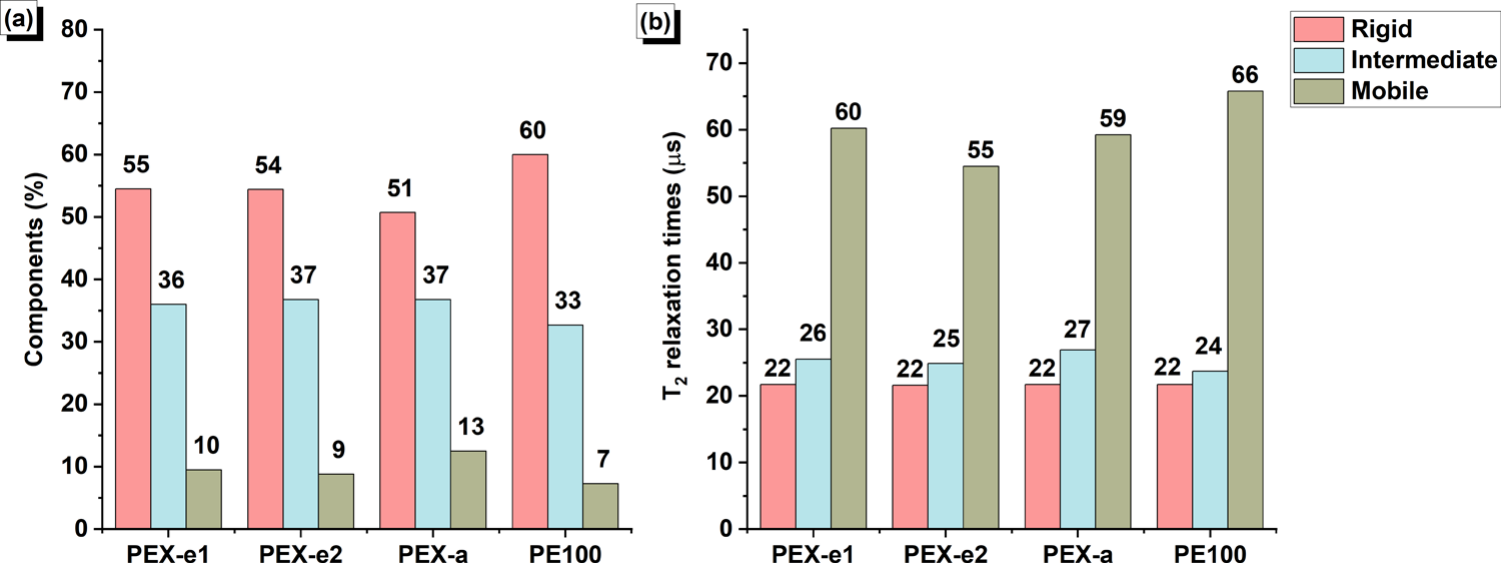
Results of the fittings for the ^1^H NMR decay showing (a) the amount of rigid (crystalline), intermediate (interphase) and mobile (amorphous) components and (b) their corresponding *T*_2_ relaxation times. (The color legend applies to both graphs.)

Quantitative assessment of phase composition and molecular mobility is crucial in material development, as these phases exhibit vastly different morphology and, consequently, have different contributions to the material’s properties. Static ^1^H NMR was used to quantify the phase composition and molecular mobility of the different pipes investigated in this study. The solid echo decay (Figure 2) was decomposed into three components, as demonstrated in Figure 3 for PEX-e2. The fitting results for all samples, presented in Figure 4 and Table 3, reveal the proportions of rigid, intermediate and mobile components, along with their corresponding *T*_2_ relaxation times.

**Table 3:** Results of ^1^H NMR Echo Decay Fitting

	rigid component (eq 2)			intermediate component (eq 3)			mobile component (eq 4)	
material	total (%)	*T*_2_ (μs)	*R* (−)	total (%)	*T*_2_ (μs)	*n* (−)	total (%)	*T*_2_ (μs)
PEX-e1	54.5	21.7	0.20	36.0	25.5	1.35	9.5	60.2
PEX-e2	54.4	21.6	0.20	36.8	24.9	1.39	8.8	54.5
PEX-a	50.7	21.7	0.21	36.8	26.7	1.35	12.5	59.2
PE100	60.0	21.7	0.21	32.7	23.7	1.24	7.3	65.8

As previously discussed, the second moment (*M*_2_) is a measure of the relative proximity of the neighboring H’s in the lattice. The calculated second moment for rigid polyethylene is 25.5 G^2^^39^ and the experimental second moment at 84 K was found to be 26.3 G^2^.^60^ The observed values shown in Table 4 vary between 25.2 and 25.6 G^2^, indicating that the resulting Abragam component is, in fact, due to the rigid part of the polymer and can be assigned to the crystalline region. As expected for the crystalline domains, the mobility within this area does not change between samples, as the measurements were performed below the crystalline melting point.

**Table 4:** Additional Parameters of the ^1^H NMR Echo Decay Fitting

material	rigid component (%) (eq 5)	*M*_2_ (G^2^) (eq 6)
PEX-e1	54.5	25.4
PEX-e2	54.4	25.2
PEX-a	50.7	25.6
PE100	60.0	25.6

PEX-a has the lowest amount of rigid components (lowest degree of crystallinity) and the highest amount of mobile components. This reduced crystallinity is likely due to its higher degree of cross-linking. In both the peroxide- and photoinitiated processes, cross-linking occurs at temperatures above the melting point, which directly influences subsequent crystallization. During cooling, the presence of cross-links hinders chain folding and close packing, leading to a lower degree of crystallinity.^7,25,54,61,62^ Accordingly, the non-cross-linked PE100 exhibits a higher proportion of rigid components compared to the other samples.

The amount of interphase material in PEX-a is roughly the same as that in PEX-e1 and PEX-e2. However, if the degree of cross-linking is higher, the mobility of the interphase region is expected to be reduced. Surprisingly, it is slightly more mobile (longer ) compared to PEX-e1 and PEX-e2. This could be attributed to the nature of the cross-links. As previously discussed, the cross-links in PEX-e are not solely composed of carbon–carbon bonds,^18^ as they are in PEX-a, which may influence the mobility of the network and, consequently, the mobility of the interphase.

Although PEX-e2 exhibited a significantly greater cross-linking density compared to PEX-e1, their phase proportions and interphase mobility are rather similar. However, the amorphous phase in PEX-e1 showed greater mobility compared to that in PEX-e2. These findings support the assumption that the cross-links are more evenly dispersed in PEX-e1 compared to PEX-e2. A more uniform distribution of cross-links within the interphase region of PEX-e1 would allow for slightly more free PE chains in the amorphous region, resulting in higher  relaxation times.

### Melting and Crystallization Behavior

**Figure 5:**
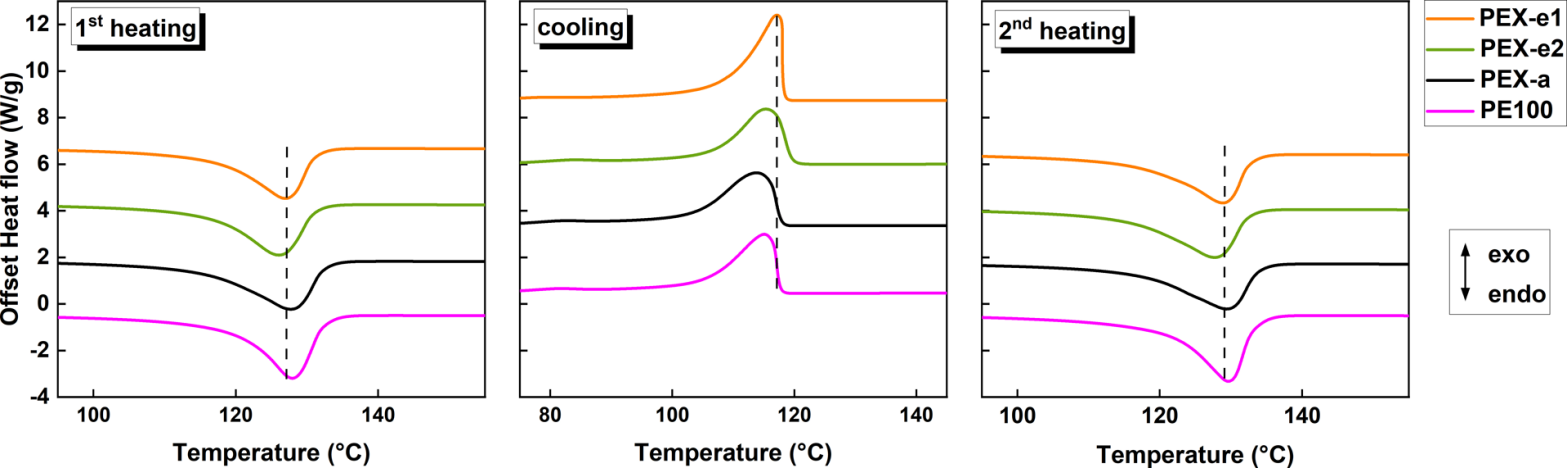
DSC curves showing first heating, cooling, and secondary heating cycles for pipe PEX and PE pipe samples.

Differential scanning calorimetry was used to assess the melting and crystallization behavior of the pipe samples. The heating and cooling curves of PEX and PE are shown in Figure 5, while the average values for the melting temperature (*T*_m_), crystallization temperature (*T*_c_), melt enthalpy (Δ*H*_m_) and crystallinity degree (*X*_c_) are listed in Table 5. All materials exhibited distinct endothermic melting peaks and well-defined crystallization peaks.

**Table 5:** Parameters Obtained from the DSC Curves of the PEX and PE Pipes^a^

material	*T*_m_^1^ (°C)	*T*_m_^2^ (°C)	*T*_c_ (°C)	Δ*H*_*m*_^1^ (J/g)	*X*_c_^1^ (%)	Δ*H*_m_^2^ (J/g)	*X*_c_^2^ (%)
PEX-e1	127 ± 1	129 ± 0	115 ± 0	153 ± 6	54.4 ± 2.4	176 ± 3	62.7 ± 1.7
PEX-e2	126 ± 0	129 ± 1	113 ± 0	180 ± 6	63.9 ± 2.5	183 ± 3	65.1 ± 1.7
PEX-a	128 ± 1	130 ± 1	114 ± 2	169 ± 3	60.0 ± 1.6	173 ± 2	61.4 ± 1.5
PE100	128 ± 1	130 ± 0	117 ± 1	203 ± 3	72.1 ± 1.9	212 ± 4	75.4 ± 2.1

a The superscripts 1 and 2 stand for first and second heating, respectively. (Average and standard deviation for three replicate runs.)

The first heating curve is typically performed to erase the samples’ thermal history. However, in this study, the first melt peak is of particular interest, as it provides information on the effect of the cross-linking process on the properties of the pipes. During the first heating, PEX-e2 displayed a slightly lower melting temperature compared to those of the other materials, indicating differences in the crystalline phase morphology. In general, lower lamellae thickness and lower crystal regularity result in lower melting temperatures.^61,63^

Notable differences in melt enthalpy, and consequently in the crystallinity degree, were observed between the first and second heating cycles. In general, all materials showed lower crystallinity () during the first heating compared to the second (), suggesting that the processing conditions negatively impacted crystallization. This effect was more pronounced in PEX-e1, which exhibited a considerably lower value of . Once the thermal history was erased and the chains were allowed to reorganize and recrystallize, the melt enthalpy increased for all of the samples. In the second heating, the differences in the melting temperature between the materials were statistically insignificant.

The crystallinity results obtained from DSC are generally inconsistent with the phase composition determined by NMR (see Section Molecular dynamics and quantitative phase composition). While both methods consistently indicate that PE100 has a higher crystallinity than the PEX samples, the crystallinity values determined by DSC are generally higher than those measured by NMR. Additionally, NMR indicated that both PEX-e pipes had similar phase compositions, whereas DSC indicated a higher crystallinity for PEX-e2, compared to PEX-e1. This discrepancy between DSC and NMR was also observed by Posadas et al.^6^ and it is likely due to differences in how each method measures crystallinity. DSC measures thermal properties, quantifying crystallinity based on the heat required to induce the melt transition. Proton NMR, on the other hand, measures the physical structure of the polymer at a molecular level, by examining the mobility and arrangement of hydrogen nuclei, distinguishing between the highly ordered crystalline region, a slightly ordered interphase, and the more mobile amorphous region.

The crystallization temperature was similar among the PEX variants. However, as shown in Figure 5, the shape of the crystallization and melting peaks varies slightly among them. PEX-e1 displays a sharper crystallization peak and a slightly narrower melting peak compared with PEX-e2 and PEX-a. These observations indicate slight differences in crystallization kinetics and crystallite size distribution.^64^ Broader peaks are often associated with the presence of imperfect crystals with wider lamellae thickness distribution.^65^ As highlighted by Qing et al.,^22^ when some segments between adjacent cross-links are long enough to fold, certain cross-links may become embedded within the crystal phase, having little effect on the crystallinity degree, but acting as defects and reducing crystal regularity.

This crystal irregularity may also contribute to the disparity between the DSC and NMR crystallinity measurements. Crystalline regions containing defects likely exhibit different mobilities, leading them to be more accurately detected as interphase by NMR. Further investigation into the morphology of the crystalline phase would be valuable to confirm this assumption. While conventional DSC provides limited insight into crystal regularity, more detailed analysis using modulated DSC^63,65^ appears to be a promising direction for future research.

### Antioxidant Content and Degradation Products

During their manufacturing, installation, and service, PE and PEX pipes can undergo thermo-oxidative and photo-oxidative degradation reactions that can lead to premature failure.^12,66^ Accordingly, antioxidant (AO) additives are incorporated into their formulations in order to prevent oxidative degradation and to extend their service time.

**Figure 6:**
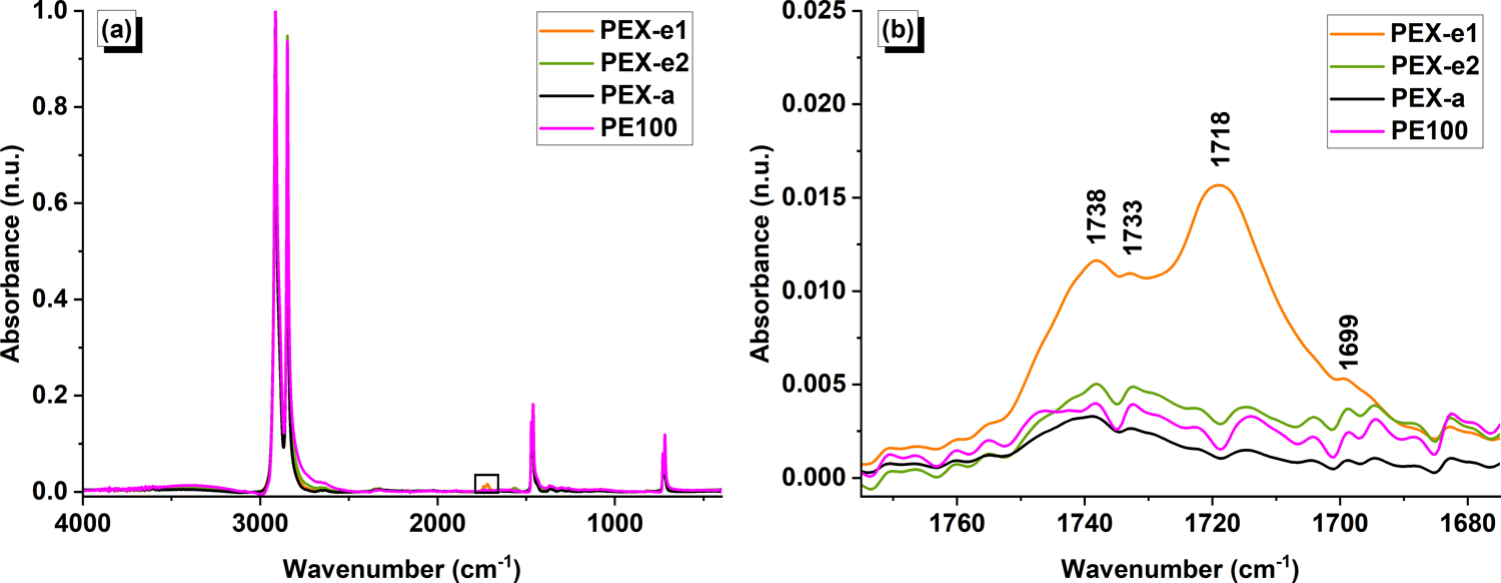
FTIR spectrum of PEX and PE pipe samples: (a) 4000–400 cm^–1^ range and (b) carbonyl region (1775–1675 cm^–1^).

FTIR spectroscopy was used to analyze the antioxidant additive content and identify chemical species that play an important role in polymer degradation. Figure 6 shows the average spectrum for each pipe material. The main absorbance peaks observed are characteristic of PE molecular vibrations. The CH_2_ asymmetric (ν_a_) and symmetric (ν_s_) stretching modes were observed at 2915 and 2847 cm^–1^, respectively. The doublet characteristic of CH_2_ scissoring mode (δ) was observed at 1472 and 1462 cm^–1^, while the rocking mode (γ_r_) peaks were identified at 730 and 719 cm^–1^. The maximum variation in peak position among materials was ±1 cm^–1^, which is lower than the experimental resolution.

The carbonyl region, featured in Figure 6b, is of specific interest for the analysis of additive content and degradation products in polyolefins.^44,66^ Particularly, the most commonly used hindered phenolic antioxidant additives for stabilization of PEX and PE, such as Irganox 1010 and Irganox 1076, contain ester linkages with characteristic absorbance peaks reported at 1738–1740 cm^–1^.^8,66−68^ Accordingly, the carbonyl peaks located at 1739 cm^–1^ in PEX-e1 and 1738 cm^–1^ in the other pipes were assigned as an ester. Additional peaks located approximately at 1733, 1718, and 1699 cm^–1^ were assigned as aldehyde,^69,70^ ketone^13,69,70^ and carboxylic acid,^12,13,69^ respectively.

**Figure 7:**
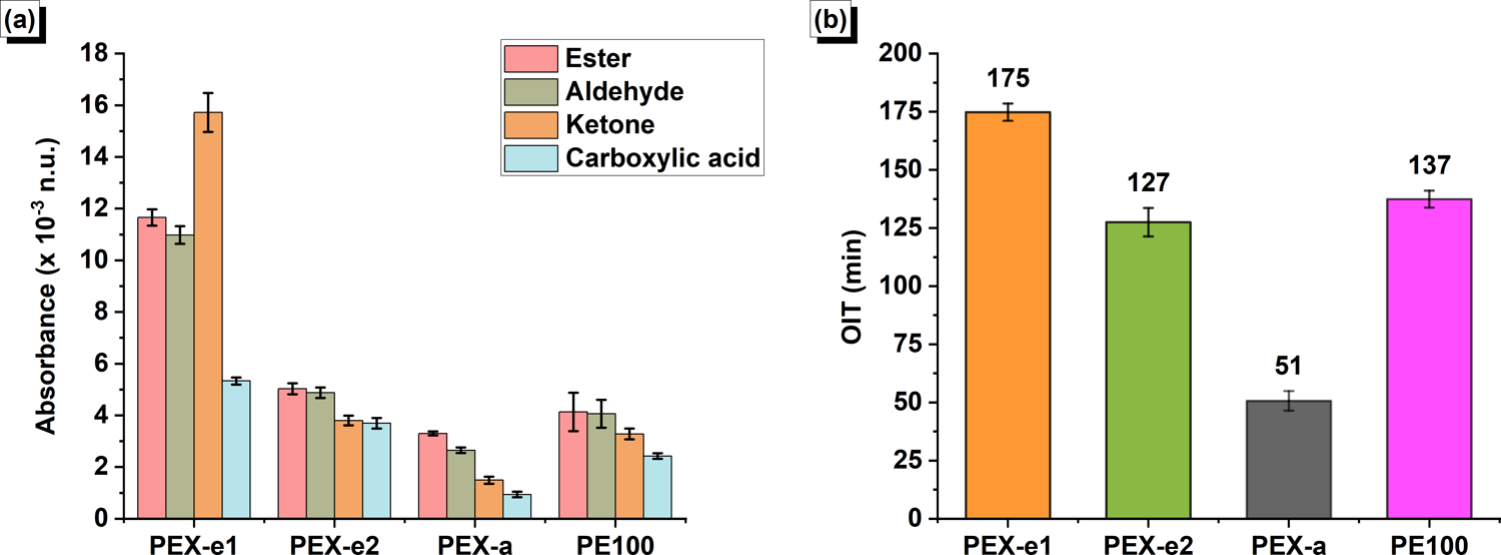
(a) Normalized absorbance of carbonyl groups and (b) OIT values for the PEX and PE pipes. (Error bars represent the standard deviation.)

Although precise quantification of additive concentration with FTIR cannot be done without a calibration curve,^44,67^ quantification of the carbonyl index and ester absorbances can provide valuable insight into the additive content among different pipes. The carbonyl index and average normalized absorbance of the carbonyl peaks detected in the spectra of the pipes are listed in Table 6, with the latter also shown in Figure 7a for a clearer visualization. PEX-e1 samples presented a significantly higher carbonyl index compared to the other pipe materials. Notably, the ester absorbance in PEX-e1 was more than twice that of the other pipes. This result confirmed the higher percentage of AO additives in the Formulation I was informed by the supplier.

**Table 6:** Carbonyl Index (CI) and Normalized Absorbance of Carbonyl Groups in the FTIR Spectra of the Pipes

material	CI ×10^–2^ (cm^–1^)	ester ×10^–3^ (n.u.)	aldehyde ×10^–3^ (n.u.)	ketone ×10^–3^ (n.u.)	carboxylic acid ×10^–3^ (n.u.)
PEX-e1	65.2 ± 1.9	11.7 ± 0.3	11.0 ± 0.3	15.7 ± 0.8	5.3 ± 0.1
PEX-e2	26.9 ± 1.5	5.0 ± 0.2	4.9 ± 0.2	3.8 ± 0.2	3.7 ± 0.2
PEX-a	13.2 ± 0.8	3.3 ± 0.1	2.7 ± 0.1	1.5 ± 0.1	0.9 ± 0.1
PE100	23.4 ± 0.9	4.1 ± 0.7	4.1 ± 0.5	3.3 ± 0.2	2.4 ± 0.1

Comparatively, PEX-a samples contained fewer ester groups, suggesting that a portion of the additives might have been consumed during processing. The PEX-a method is known for its potential to overconsume antioxidant (AO) stabilizers,^71^ as cross-linking occurs at elevated temperatures (170–190 °C^72^) in the presence of organic peroxides. In contrast, the PEX-e process occurs at lower temperatures (around 135 °C) and does not involve peroxide agents. However, oxidation has been reported to occur during photo-cross-linking.^23^ In fact, as evidenced in Figure 7a, the absorbance of ketones, aldehydes, and carboxylic acids, which are known oxidation products,^47,67^ was also comparatively higher in the PEX-e1 samples. These findings indicate that despite the increased amount of AO additives, the PEX-e1 pipe underwent some level of degradation during processing.

Although the extrusion of polyolefins occurs in an oxygen-poor environment, it has been proven that numerous degradation reactions can take place during the high-temperature processing of polyethylene.^73−75^ Various factors influence the extent of these reactions, including the chemical structure of the polymer, type and amount of catalyst residues, the composition of the additive package, as well as the processing conditions, such as temperature, the concentration of oxygen diffused into the sample, the intensity of shear forces, etc.^73,74^ In the case of PEX, the cross-linking process can also play an important role in degradation.^71^ For PEX-e, the presence of a certain amount of unreacted residual photoinitiator and its photolytic products may also influence the stability of the cross-linked polymer.^21^

Oxidation induction time (OIT) is one of the most frequently used methods for assessing the oxidative stability and the performance of AOs in polyolefin pipes.^66,76^ The OIT values for all pipe samples were measured and are presented in Figure 7b. Notably, PEX-e1 exhibited significantly higher OIT values compared to the other materials, which can be expressed by its higher AO content. In contrast, the average OIT value obtained for PEX-a was considerably lower than that of the other materials, which is consistent with the FTIR observations.

Previous studies have reported correlations between OIT values and concentration of primary AO additives.^66,77^ However, this correlation was not observed in some of the pipes. Although the carbonyl index and ester absorbance detected in the FTIR spectra of PE100 and PEX-e2 were relatively low, the OIT values measured for these materials were significantly high (above 120 min). These findings underline that the values of the OIT are influenced not only by the concentration of primary AOs but also by the effectiveness of the entire additive package. Certain combinations of primary and secondary AOs have been shown to produce a synergistic effect, reducing the total amount of primary AOs required for stabilization.^68,73,78^

### Thermal Degradation

**Figure 8:**
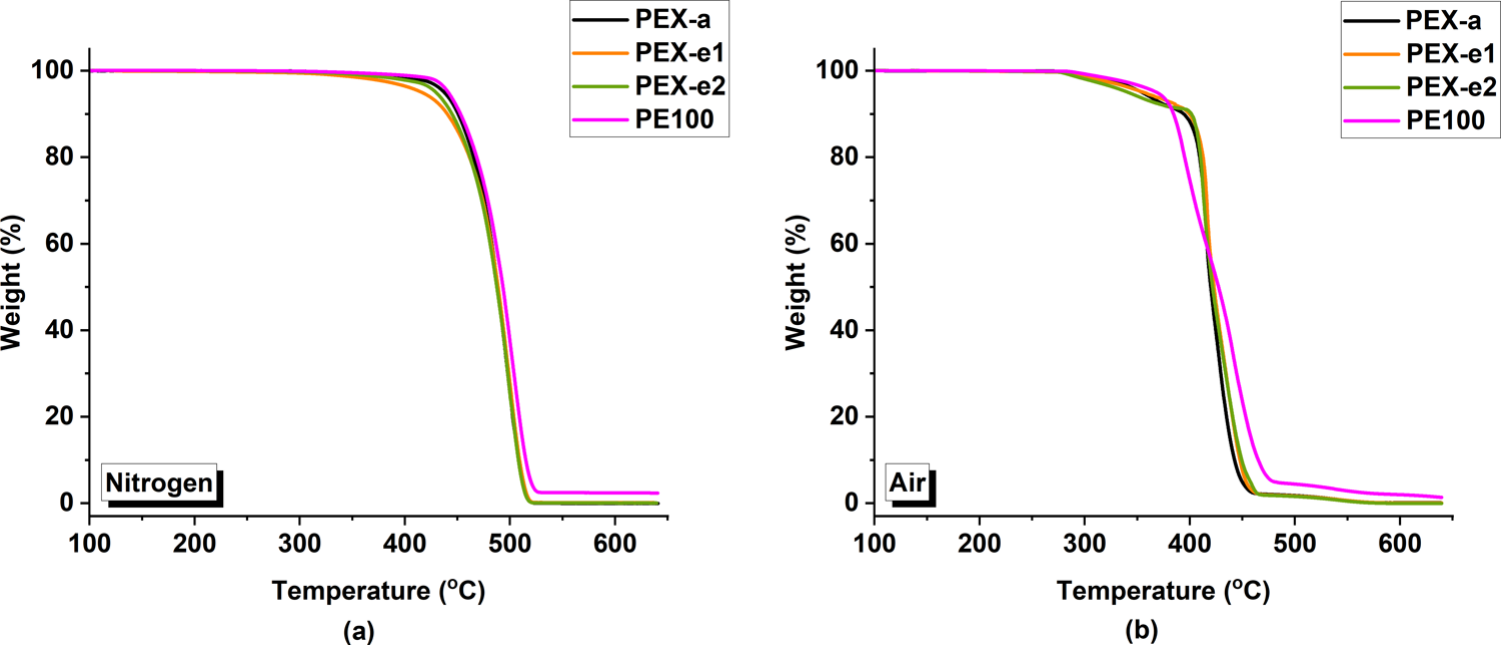
TG curves of pipe samples under (a) nitrogen and (b) air atmospheres. (One representative curve was chosen for each material based on the median values from three replicate tests.)

Thermogravimetric studies were conducted under nitrogen and air atmospheres to evaluate the thermal degradation of the pipes. Figure 8 shows the TG curves for the PEX and PE pipes in nitrogen and air atmospheres. A final residue of 2.3 wt % was observed for PE100, which was attributed to the carbon black additive. All other samples reached less than 0.1 wt % residue at 650 °C. The relative thermal stability of the materials can be analyzed using the parameters listed in Table 7.

**Table 7:** Thermal Degradation Parameters for Pipes Were Obtained from the TG Curves

purge gas	material	*T*_5_ (°C)	*T*_50_ (°C)	*T*_95_ (°C)	*T*_max_ (°C)
nitrogen					
	PEX-e1	416 ± 3	490 ± 4	514 ± 2	499 ± 3
	PEX-e2	433 ± 6	491 ± 6	514 ± 3	501 ± 4
	PEX-a	445 ± 10	492 ± 5	514 ± 3	499 ± 5
	PE100	451 ± 15	497 ± 5	519 ± 1	506 ± 1
air					
	PEX-e1	354 ± 2	424 ± 1	451 ± 2	419 ± 2
	PEX-e2	340 ± 2	425 ± 3	457 ± 2	418 ± 4
	PEX-a	351 ± 3	421 ± 1	452 ± 2	418 ± 2
	PE100	360 ± 6	425 ± 3	487 ± 8	425 ± 17

Under a nitrogen atmosphere, the materials degraded in a single step beginning around 430 °C and reaching 95% of weight loss at approximately 515 °C. Surprisingly, the degradation of PE100 occurred at a slightly higher temperature range, indicating higher thermal resistance when compared to the PEX samples. Among the PEX pipes, PEX-a had the highest average *T*_5_ value at 445 °C, while PEX-e1 had the lowest value at 416 °C, indicating that PEX-a can withstand higher temperatures under inert conditions. However, this disparity was less prominent under atmospheric conditions.

**Figure 9:**
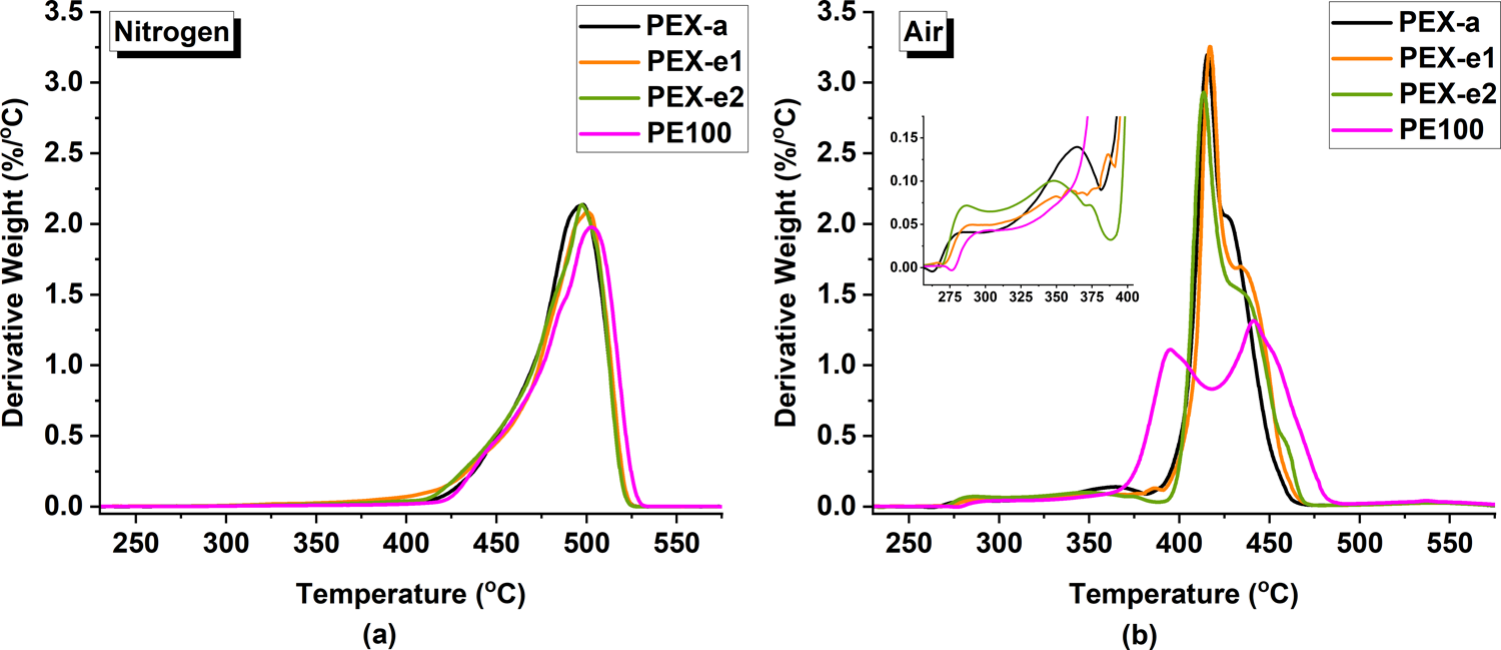
DTG curves of pipe samples under (a) nitrogen and (b) air atmospheres. (One representative curve was chosen for each material based on the median values from three replicate tests.)

As expected, the degradation range shifts to lower temperatures under an air atmosphere, generally occurring between 350 and 420 °C approximately. Additional effects of the purge gas can be observed in the DTG curves shown in Figure 9. Under a nitrogen flow, a single degradation peak was observed for all materials. According to Peterson et al.,^79^ in an inert atmosphere, the degradation mechanism of PE involves both chain scission and chain branching, which occur simultaneously, leading to a single mass loss step. Findings reported by Du et al.^80^ indicate that cross-linking and chain scission are two competitive reactions that occur concurrently within a single degradation stage during the pyrolysis of PEX.

Conversely, under an air atmosphere, the degradation of PE and PEX occurs through numerous degradation steps due to the presence of oxygen and high temperatures. This leads to various thermo-oxidative degradation reactions with chain radicals that accelerate the degradation process.^48,79,81^ At least three degradation stages were observed for PE and PEX in air: the first stage consisted of small irregular degradation peaks in the 270–400 °C region, as highlighted in Figure 9b. The second stage consisted of a well-defined degradation peak at 395 °C for PE100 and 418 °C for PEX. The third stage consisted of another peak, which appeared as a shoulder between 420 and 430 °C for PEX, while for PE100 it involved a defined peak around 440 °C.

The differences observed in Figure 9 indicate that the kinetics of PE degradation in air changes in the presence of cross-links. For PEX, the main degradation occurred in stage two, with a very intense and sharp peak, indicating faster degradation compared to PE100. For the latter, the degradation transpired over a wider temperature range throughout stages two and three. A possible explanation for the faster degradation in PEX might be the presence of weak link sites, such as peroxides, carbonyl groups and unsaturated structures, induced by the cross-linking process.^79,81^ Further investigations of the chain morphology and degradation kinetics could better elucidate these differences.

### Thermo-Mechanical Properties

**Figure 10:**
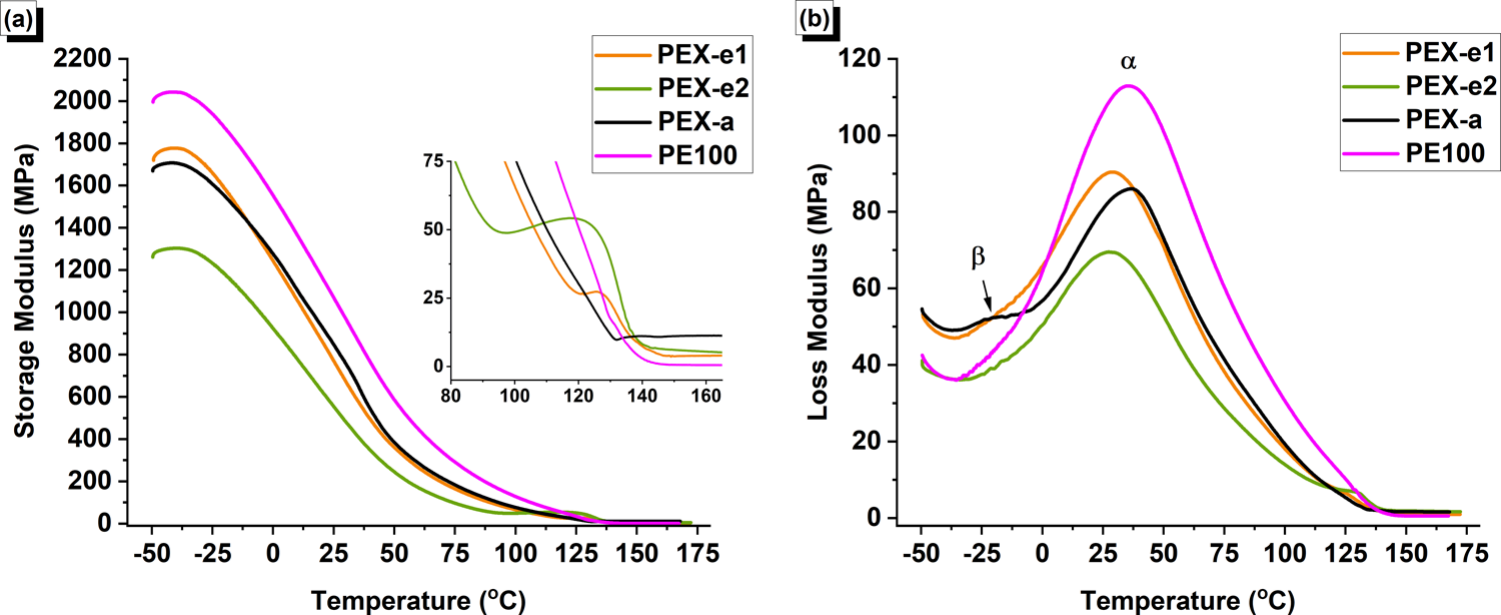
(a) Storage modulus and (b) loss modulus of PEX and PE pipe samples. (One representative curve was chosen for each material based on the median values from three replicate tests.)

The dynamic mechanical properties of the PEX and PE pipes over the temperature range of −50 to 170 °C are shown in Figure 10. Significant differences in storage modulus (*E’*) were observed among the pipes, with these differences being more pronounced at lower temperatures, as detailed in Table 8. Below the melting point, the storage modulus is strongly influenced by the crystalline morphology.^49,50^ In general, higher crystallinity leads to higher mobility restriction and, consequently, higher storage modulus. Accordingly, the higher *E’* values exhibited by PE100 below the melting point can be attributed to its higher degree of crystallinity. In general, cross-linked PE exhibits lower stiffness compared to non-cross-linked PE due to the negative impact of the cross-linked network on crystallinity.^64^ However, the differences observed among the PEX pipes suggest that the morphology of the cross-link network also plays a critical role in determining their mechanical properties.

**Table 8:** Parameters Obtained from the DMA Curves of PEX and PE Pipes (Average and Standard Deviation for Three Replicate Runs)

material	*E′* at 0 °C (MPa)	*E′* at 23 °C (MPa)	*E′* at 90 °C (MPa)	*E′* at 160 °C (MPa)	*T*_α_ (°C)	*E”* at T_α_ (MPa)
PEX-e1	1213 ± 44	791 ± 42	90 ± 7	4 ± 0	29 ± 2	88 ± 2
PEX-e2	920 ± 27	584 ± 21	54 ± 5	6 ± 1	27 ± 1	68 ± 1
PEX-a	1251 ± 40	868 ± 15	111 ± 0	12 ± 1	35 ± 2	85 ± 2
PE100	1510 ± 65	1098 ± 78	192 ± 25	1 ± 0	38 ± 4	110 ± 4

As shown in Figure 10a, PEX-a and PEX-e1 exhibited similar stiffness overall, with PEX-a having a slightly lower storage modulus at around −50 °C. This difference can be attributed to the lower proportion of the rigid phase, i.e., reduced crystallinity, as identified by NMR analysis. As the temperature increases, molecular motion becomes more pronounced, leading to a continuous decrease in the storage modulus for all materials. However, PEX-a showed a slower decrease in *E’* with temperature, compared to PEX-e1, maintaining slightly higher *E’* values above −5 °C. This more gradual decline is likely due to the higher cross-linking density in PEX-a. In general, shorter chain segments between cross-links, as evidenced by the lower *M*_c_ values, inhibit chain movement, contributing to a stiffer network.^49^

Despite having a similar amount of rigid phase as PEX-e1, PEX-e2 unexpectedly exhibited a significantly lower storage modulus below melting. This suggests that other morphological factors, such as the dispersion of cross-links, are crucial in determining mechanical properties. Indeed, Shen et al.^82^ found that improving cross-link dispersion can have a similar effect on the elastic modulus as increasing the cross-link density. For polymers with the same cross-link density, greater cross-link dispersion resulted in a notably higher storage modulus, while more aggregated networks led to reduced stiffness.^82^ Thus, the lower stiffness observed in PEX-e2 could be explained by a less uniform distribution of cross-links in this material.

Once the material surpasses its melting point, it becomes fully amorphous, and the storage modulus reaches a minimum and stabilizes at a plateau. This plateau, known as the rubbery region, is proportional to the density of cross-links and molecular entanglements.^49,50^ As shown in the zoomed-in section of Figure 10a, the storage modulus of the PEX pipes does not exhibit a continuous decrease at melting, as seen with non-cross-linked PE. Instead, a step occurs before the modulus stabilizes at the plateau, with this effect being more pronounced in PEX-e2 and less pronounced at PEX-a. Xie and Li^50^ observed a similar phenomenon in ultrahigh molecular weight polyethylene, attributing it to a highly entangled network. In the case of PEX, this behavior could be related to the cross-linked network structure. It can be speculated that the larger step observed in PEX-e2 compared to the other PEX pipes is due to higher chain entanglement and aggregation of cross-links in this material.

The loss modulus (*E′*) is a measure of the energy dissipated as heat during the deformation cycle. The loss modulus curves reveal relevant information about the mechanical relaxations occurring over different temperature ranges. Polyethylene displays three transitions conventionally designated as α, β, and γ relaxations.^83^ The γ transition is usually observed in the range −150 to −120 °C, the β transition in the range −30 to 10 °C and the α transition is usually found between 30 and 120 °C.^83,84^

The α-relaxation is assigned to the motion or deformation of chain units within the crystalline regions.^85,86^ In the *E*″ curve, this transition was observed at lower temperatures for both PEX-e, compared to PEX-a and PE100, respectively. Differences in the shape of the α peak were also evident. The main factor governing *T*_α_ in linear polyethylene has been found to be lamellar thickness.^83,85^ Popli et al.^83^ also noted that the morphology of the spherulites and the interfacial structure might have a significant effect on the α transition. Unfortunately, no literature regarding the effect of cross-links on the α transition was found. However, the values obtained in the present study suggest that the different morphology formed in the PEX-e cross-linking process shifts the α relaxation to lower temperatures.

A weak β transition peak was observed around −22 °C for PEX-a. This transition was not resolved for the other materials. β relaxation has been attributed to the relaxation of chain units located at the amorphous and interfacial regions and is usually hardly resolved for linear PE.^83^

## Conclusions

The PEX pipes produced using a novel photo-cross-linking method (PEX-e) were characterized in detail. Two formulations, PEX-e1 and PEX-e2, were analyzed and compared to peroxide-cross-linked polyethylene (PEX-a) and non-cross-linked bimodal polyethylene (PE100) pipes. Both PEX-e formulations achieved high cross-linking degrees (above 70%). However, significant differences in cross-link density and molecular weight between cross-links were noted, suggesting PEX-e2 had a more aggregated cross-linked network.

Static ^1^H NMR spectroscopy provided insights into the phase composition and molecular mobility of the different pipes. A three-component model was used to fit the solid echo decay data, distinguishing contributions from the crystalline phase, interphase, and amorphous region. As expected, the PEX samples exhibited a lower degree of crystallinity compared to PE100. Among the PEX variants, the PEX-e samples showed a slightly higher proportion of the rigid component compared to PEX-a, which was attributed to the higher gel content in PEX-a. Differences in the interphase relaxation times suggested that the bonds formed in the PEX-e process create a more rigid network with reduced molecular mobility compared to the carbon–carbon bonds formed in the PEX-a process.

FTIR analysis revealed a lower antioxidant (AO) content in PEX-a, suggesting overconsumption of stabilizers during processing. This was supported by the significantly low oxidation induction time (OIT) values observed in PEX-a. Both FTIR and OIT analyses indicated higher AO content in PEX-e1 compared to the other pipes. However, the PEX-e1 spectrum also showed signs of oxidation with increased absorbance of ketones, aldehydes, and carboxylic acids. This suggests that despite the higher AO content, some oxidation occurred during processing. Interestingly, both PEX-e2 and PE100 also showed high OIT values despite having relatively low primary AO content, suggesting potential synergistic effects from their additive package formulations.

Differential scanning calorimetry (DSC) revealed that PEX-e2 had a slightly lower melting temperature, indicating a reduced crystal regularity, which was further supported by broader crystallization and melting peaks. The thermal degradation of the materials, as assessed by TGA, followed similar trends in an inert atmosphere but showed significant differences in the air. Under a nitrogen atmosphere, all materials are degraded in a single step, whereas in air, degradation occurs in multiple steps. Among the PEX samples, PEX-a had the highest onset temperature under nitrogen (445 °C), while PEX-e1 had the lowest (416 °C), suggesting that PEX-a can withstand higher temperatures under inert conditions. However, this disparity was less prominent under atmospheric conditions.

Dynamic mechanical analysis (DMA) revealed significant differences in thermo-mechanical behavior. Below the melting point, PE100 exhibited a significantly higher storage modulus, which was attributed to its higher degree of crystallinity. However, the differences observed among the PEX pipes suggested that crystallinity alone was not the only factor influencing dynamic mechanical properties; the morphology of the cross-link network also played a critical role. The higher cross-linking degree in PEX-a resulted in a less steep decline in storage modulus (*E’*) with temperature. Additionally, the lower storage modulus observed in PEX-e2 below the melting point further supported the idea of cross-link aggregation in this pipe.

In conclusion, PEX-e pipes show promise for high-temperature borehole thermal energy storage systems, as both formulations investigated exhibited high degrees of cross-linking and robust OIT values. For PEX-e1, the storage modulus was comparable in magnitude to that of commercially established PEX-a. However, the presence of oxidation products in PEX-e1 highlights the importance of refining the formulation, particularly in the selection and concentration of stabilizing additives. This optimization is a critical direction for future work to enhance long-term stability. Additionally, further investigation into processing conditions, photoinitiators, and cross-linking coagents could play an important role in achieving PEX-e pipes with properties comparable to, or exceeding, those of PEX-a.

## Supporting Information Available

FTIR spectroscopy data processing (PDF)
