# Supplementary material for: Characterization of Photo-Cross-Linked Polyethylene Pipes for Geothermal Energy Storage
Source: ACS Omega. 2025 Jan 3;10(1):1748–60. doi: 10.1021/acsomega.4c09896 (PMC11740129; doi:10.1021/acsomega.4c09896)
Supplement: Supplementary file 1 — ao4c09896_si_001.pdf [file ao4c09896_si_001.pdf]

Supporting information: FTIR spectroscopy data processing

Giulia Simão de Sousa,[*,†,‡] Andrew Root,[¶] Ivo Heinmaa,[§] Adib Kalantar Mehrjerdi,[†,‖] José Roberto Moraes d'Almeida,[‡] and Mikael Skrifvars[†]

†*Swedish Centre for Resource Recovery, Faculty of Textiles, Engineering and Business, University of Borås, Sweden*

‡*Department of Chemical and Materials Engineering, Pontifical Catholic University of Rio de Janeiro, Brazil*

¶*MagSol, Finland*

§*National Institute of Chemical Physics and Biophysics, Estonia*

‖*MuoviTech AB, Sweden*

E-mail: giulia.simao_de_sousa@hb.se

Description

This document provides supporting information on the processing of FTIR spectroscopy data related to the publication 'Characterization of photo-crosslinked polyethylene pipes for geothermal energy storage.' The first section discusses three different approaches to baseline correction. The second section includes the Python script developed for the pre-processing and quantitative analysis of FTIR data.

FTIR baseline correction

Reliable quantitative analysis of FTIR spectra requires pre-processing of the spectral data, specifically through baseline correction. In this study, the spectra of the pipes were notably tilted due to curvature effects, as the ATR measurements were taken on the inner surface of the pipes. For example, Figure S1 shows the spectra of three replicates from the PEX-e2 pipe, demonstrating this effect and its variability among replicates of the same pipe.

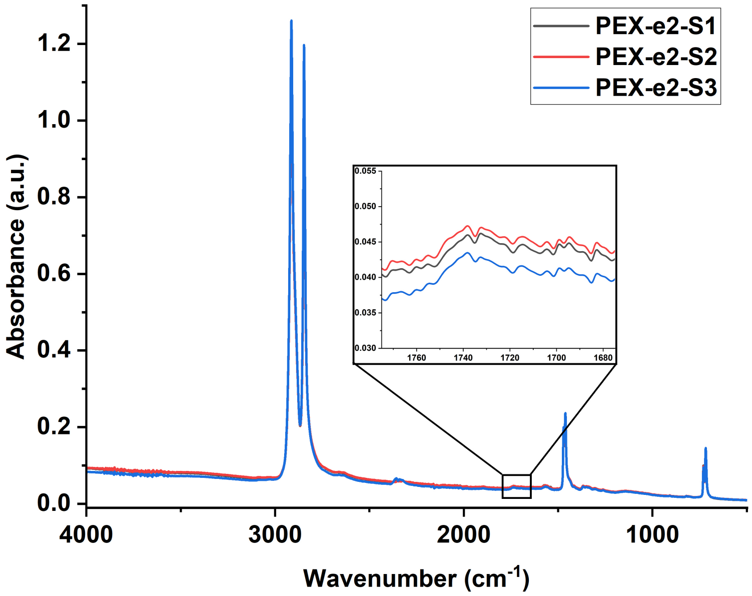

Fig. S1: FTIR spectra of three replicate samples of pipe PEX-e2.

The spectra were recorded using OMNIC software, which includes an "Automatic Baseline Correction" option. However, this option does not provide control over the baseline correction parameters, and no documentation was found regarding the methodology behind this function. To ensure a more consistent baseline correction across all replicates and materials, the authors chose to automate the analysis using Python.

Two approaches were tested: a polynomial fit[1] using the `baseline` function from the *Peakutils* library, and asymmetric least squares smoothing (ALSS)[2] using the `irfpy` library.[3] Figure S2 presents a comparison of the spectra before and after baseline correction with OMNIC software, a 4th order polynomial fit in Python, and the ALSS fit in Python. As shown in Figure S2.b, the baseline correction method significantly affects peak intensity.

Among the methods tested, the ALSS fit produced the most favorable results and was therefore adopted for processing the FTIR data reported in the publication.

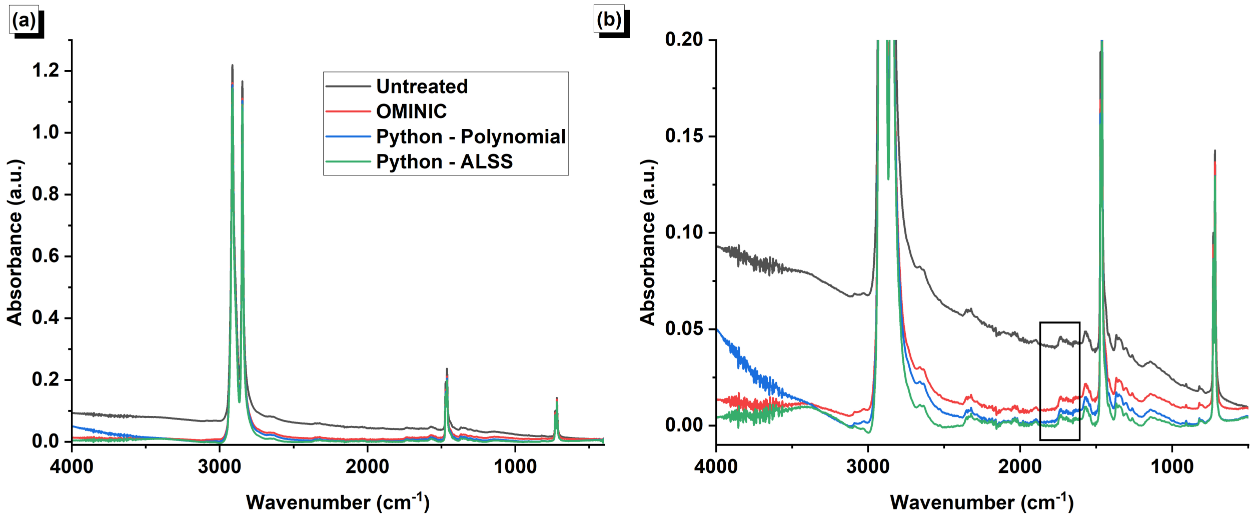

Fig. S2: (a) Full FTIR spectra and (b) zoom of a PEX-e2 sample highlighting the effect of the baseline correction on the carbonyl region. (The color legend applies for both graphs).

Python code for quantitative FTIR spectroscopy analysis

A Python script was developed to pre-process and analyze FTIR absorbance data from multiple spectra recorded at the same resolution and range. The pre-processing steps include baseline correction using ALSS and normalization with an internal standard. After pre-processing, the spectra are further analyzed by identifying peaks, calculating the area of the carbonyl region, and generating an average spectrum.

Importing

The first part of the code imports all the necessary Python libraries and imports the Excel file containing spectra data from three replicates. The files and figures were named in a consistent way to facilitate running the script for a new data set by simply replacing all the data files names with the *Find and Replace* function (ctrl+F), as shown in Figure S3.

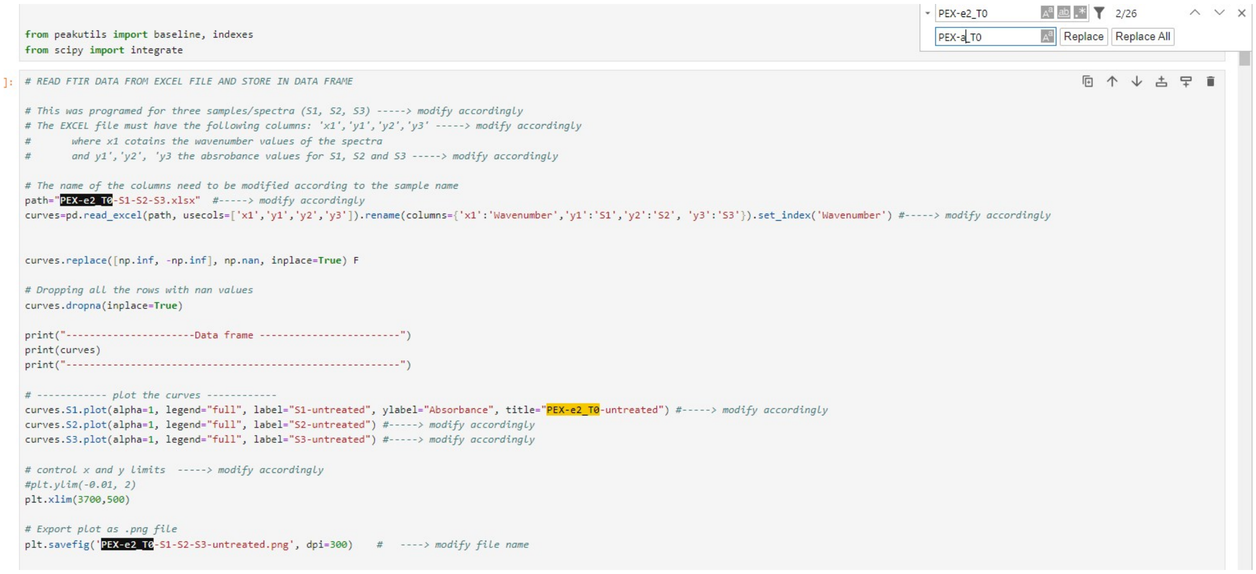

Fig. S3: Screen shot showing the use of ctrl+F to find the data file names and replace with the name of the next set to be analyzed.

```
# CODE FOR FTIR DATA PROCESSING AND ANALYSIS
# Giulia Simao de Sousa


# This code was created to simultaneously pre-process and analyse FTIR
↪   absorbance data of multiple spectra recorded with the same
↪   resolution/range
# for instance three spectra with 4 cm-1 resolution and within 400-4000
↪   cm-1 (same number of points for all spectra).
# It accepts an EXCEL file with raw FTIR data (x= wavenumber, y=
↪   absorbance) and can perform pre-processing of the data, find peaks and
↪   calculate
# area of specific regions.


# Pre-processing steps:
#     Baseline correction
```

```
#     Normalization by internal standard

# Find peaks:
#     Locates peaks in the whole spectra or within specific regions and
↪   exports the results as .csv files

# Calculate area of region
#     Numerical integration of y (absorbance) in determined dx (wavenumber
↪   interval)
#     This was meant for calculation of carbonyl index by the area of the
↪   carbonyl region but can be modified accordingly for peak areas as
↪   well

import numpy as np
import seaborn as sns
import matplotlib.pyplot as plt
import pandas as pd
import statistics
from scipy.stats import norm
import matplotlib.pyplot as pl
from scipy import sparse
from scipy.sparse.linalg import spsolve
from scipy.linalg import cholesky
from peakutils import baseline, indexes
from scipy import integrate
```

```
# READ FTIR DATA FROM EXCEL FILE AND STORE IN DATA FRAME

# This was programed for three samples/spectra (S1, S2, S3) -----> modify
↪  accordingly
# The EXCEL file must have the following columns: 'x1','y1','y2','y3'
↪  -----> modify accordingly
#       where x1 cotains the wavenumber values of the spectra
#       and y1','y2', 'y3 the absrobance values for S1, S2 and S3 ----->
↪  modify accordingly

# The name of the columns need to be modified according to the sample
↪  name
path="PEX-e2_T0-S1-S2-S3.xlsx"  #-----> modify accordingly
curves=pd.read_excel(path,
↪  usecols=['x1','y1','y2','y3']).rename(columns={'x1':'Wavenumber','y1':'S1','y2':'S2[illegible]
↪  'y3':'S3'}).set_index('Wavenumber') #-----> modify accordingly


curves.replace([np.inf, -np.inf], np.nan, inplace=True)

# Dropping all the rows with nan values
curves.dropna(inplace=True)

print("----------------------Data frame ------------------------")
print(curves)
print("---------------------------------------------------------")
```

```
# ------------ plot the curves ------------
curves.S1.plot(alpha=1, legend="full", label="S1-untreated",
↪ ylabel="Absorbance", title="PEX-e2_T0-untreated") #-----> modify
↪ accordingly
curves.S2.plot(alpha=1, legend="full", label="S2-untreated") #----->
↪ modify accordingly
curves.S3.plot(alpha=1, legend="full", label="S3-untreated") #----->
↪ modify accordingly

# control x and y limits  -----> modify accordingly
#plt.ylim(-0.01, 2)
plt.xlim(3700,500)

# Export plot as .png file
plt.savefig('PEX-e2_T0-S1-S2-S3-untreated.png', dpi=300)    #   ---->
↪ modify file name
```

Baseline correction

The second part of the code calculates baseline and subtracts it from the data.

```
# PRE-PROCESSING: BASELINE CORRECTION
#function that calculates baseline with Asymmetric Least Squares
↪ Smoothing

def als(y, lam, p, itermax):
    r"""
```

```
Implements an Asymmetric Least Squares Smoothing
baseline correction algorithm (P. Eilers, H. Boelens 2005)

Baseline Correction with Asymmetric Least Squares Smoothing
based on https://github.com/vicngtor/BaySpecPlots

Baseline Correction with Asymmetric Least Squares Smoothing
Paul H. C. Eilers and Hans F.M. Boelens
October 21, 2005

Description from the original documentation:

Most baseline problems in instrumental methods are characterized by a
↪ smooth
baseline and a superimposed signal that carries the analytical
↪ information: a series
of peaks that are either all positive or all negative. We combine a
↪ smoother
with asymmetric weighting of deviations from the (smooth) trend get an
↪ effective
baseline estimator. It is easy to use, fast and keeps the analytical
↪ peak signal intact.
No prior information about peak shapes or baseline (polynomial) is
↪ needed
by the method. The performance is illustrated by simulation and
↪ applications to
```

```
real data.


Inputs:
    y:
        input data (i.e. chromatogram of spectrum)
    lam:
        parameter that can be adjusted by user. The larger lambda is,
        the smoother the resulting background, z
    p:
        wheighting deviations. 0.5 = symmetric, <0.5: negative
        deviations are stronger suppressed
    itermax:
        number of iterations to perform
Output:
    the fitted background vector

"""
L = len(y)
D = sparse.csc_matrix(np.diff(np.eye(L), 2))
w = np.ones(L)
for i in range(itermax):
    W = sparse.spdiags(w, 0, L, L)
    Z = W + lam * D.dot(D.transpose())
    z = spsolve(Z, w*y)
    w = p * (y > z) + (1-p) * (y < z)
```

```
    return z


lam = 1e9
p = 0.005
itermax = 10


#creating baseline for each spectra
S1_baseline = als(curves.S1, lam, p, itermax)
S2_baseline = als(curves.S2, lam, p, itermax)
S3_baseline = als(curves.S3, lam, p, itermax)


#subtracting baseline
S1_sub=(curves.S1 - S1_baseline)
S2_sub=(curves.S2 - S2_baseline)
S3_sub=(curves.S3 - S3_baseline)


# Creates data frame with the baseline corrected spectra that can be
↪  exported later as .csv file
corrected_frame = {'S1_bsl': S1_sub,
                   'S2_bsl': S2_sub,
                   'S3_bsl': S3_sub
                  }
baseline_corrected_curves = pd.DataFrame(corrected_frame)


print(baseline_corrected_curves)
```

```
#CREATING A AVERAGE SPECTRA OF S1, S2, S3
Sm_sub =  baseline_corrected_curves.mean(axis=1, skipna=True)



S3_sub.plot(alpha=0.6, legend="full", label="corrected spectrum")
↪  #corrected
curves.S3.plot(alpha=1, legend="full", label="untreated spectrum",
↪  ylabel="Absorbance", title="PEX-e2_T0-baseline-correction-procedure")
↪  #untreated
sns.lineplot(y=S3_baseline, x=curves.index,  alpha=0.5,label="baseline")
↪  #baseline
# control x and y limits  -----> modify accordingly
plt.ylim(-0.01, 0.7)
plt.xlim(4000,500)
# Export plot as .png file
plt.savefig('PEX-e2_T0-S1-S2-S3-baseline-correction.png', dpi=300)    #
↪  ----> modify file name
```

Normalization

The baseline corrected spectra were normalized by a internal standard: the absorbance of the CH_2 stretching at 2915 cm^{-1}.

```
# PRE-PROCESSING: NORMALIZATION
# Normalization of FTIR data if often performed using a specific peak
↪  absorbance as an internal reference.
# In this case the absorbance of the 2914cm-1 peak was the internal
↪  reference
```

```
# Finding the intensity of the reference: function that finds peaks in
↪  spectra and returns the indexes (location of peaks in data frame)
mdist=1
thres=0.3 # threashold for peak detection -----> modify accordingly
p1 = indexes(S1_sub.values,
             min_dist=mdist,
             thres=thres
            )
p1
p2 = indexes(S2_sub.values,
             min_dist=mdist,
             thres=thres
            )
p2

p3= indexes(S3_sub.values,
             min_dist=mdist,
             thres=thres
            )
p3
pm= indexes(Sm_sub.values,
             min_dist=mdist,
             thres=thres
            )
pm
```

```
# Internal reference limits (as the values can vary a bit between
↪  spectra)
ref_lim_inf_1 = 2910 # -----> modify accordingly
ref_lim_sup_1 = 2920  # -----> modify accordingly

# Retrieves the desired peak
reference_peak_S1 = S1_sub.iloc[p1][S1_sub.iloc[p1].index > ref_lim_inf_1]
reference_peak_S1 =  reference_peak_S1[reference_peak_S1.index <
↪  ref_lim_sup_1]
reference_peak_S2 = S2_sub.iloc[p2][S2_sub.iloc[p2].index > ref_lim_inf_1]
reference_peak_S2 =  reference_peak_S2[reference_peak_S2.index <
↪  ref_lim_sup_1]
reference_peak_S3 = S3_sub.iloc[p3][S3_sub.iloc[p3].index > ref_lim_inf_1]
reference_peak_S3 =  reference_peak_S3[reference_peak_S3.index <
↪  ref_lim_sup_1]
reference_peak_Sm = Sm_sub.iloc[pm][Sm_sub.iloc[pm].index > ref_lim_inf_1]
reference_peak_Sm =  reference_peak_Sm[reference_peak_Sm.index <
↪  ref_lim_sup_1]

print("--- Internal refence absorbance ---")
print('S1 absorbance-ref:',reference_peak_S1.iloc[0])
print('S2 absorbance-ref:',reference_peak_S2.iloc[0])
print('S3 absorbance-ref:',reference_peak_S3.iloc[0])
print("----------------------------------")
```

```
#   Normalizes the baseline corrected spectra
S1_norm=S1_sub.div(reference_peak_S1.iloc[0])
S2_norm=S2_sub.div(reference_peak_S2.iloc[0])
S3_norm=S3_sub.div(reference_peak_S3.iloc[0])
Sm_norm=Sm_sub.div(reference_peak_Sm.iloc[0])

# Creates data frame with the normalized spectra that can be exported
↪   later as .csv file
norm_frame = {'S1-normalized': S1_norm,
              'S2-normalized': S2_norm,
              'S3-normalized': S3_norm,
              'Mean-normalized':Sm_norm
             }
normalized_curves = pd.DataFrame(norm_frame)
print(normalized_curves)
```

Finding peaks

Since the focus was on identifying peaks within the carbonyl region, a subset of the data was first created, followed by the application of the `indexes` function.

```
# FIND PEAKS WITHIN SPECIFIC REGION OF SPECTRA - in this case Carbonyl
↪   region (1675-1775cm-1)

#Creating subsets with spectral regions of interest
# SUBSET_1 - Region range = CARBONYL
region_lim_inf_1 = 1675.0
region_lim_sup_1 = 1775.0
```

```
S1_subset_1 = S1_norm[S1_norm.index > region_lim_inf_1]
S1_subset_1 = S1_subset_1[S1_subset_1.index < region_lim_sup_1]
S2_subset_1 = S2_norm[S2_norm.index > region_lim_inf_1]
S2_subset_1 = S2_subset_1[S2_subset_1.index < region_lim_sup_1]
S3_subset_1 = S3_norm[S3_norm.index > region_lim_inf_1]
S3_subset_1 = S3_subset_1[S3_subset_1.index < region_lim_sup_1]
Sm_subset_1 = Sm_norm[Sm_norm.index > region_lim_inf_1]
Sm_subset_1 = Sm_subset_1[Sm_subset_1.index < region_lim_sup_1]



carbonyl_frame= {'curve_S1': S1_subset_1,
                 'curve_S2': S2_subset_1,
                 'curve_S3': S3_subset_1,
                  'mean_curve': Sm_subset_1
                }


carbonyl_region = pd.DataFrame(carbonyl_frame)


# parameters
mdist=1
thres=0.2  #modify this to change threashold/sensibility used


# searches for peaks and gets index values (location of peaks in data
↪   frame)
pf1 = indexes(S1_subset_1.values,
```

```
              min_dist=mdist,
              thres=thres
             )
pf1
pf2 = indexes(S2_subset_1.values,
              min_dist=mdist,
              thres=thres
             )
pf2

pf3 = indexes(S3_subset_1.values,
              min_dist=mdist,
              thres=thres
             )
pf3

peaks_S1 = pd.Series(S1_subset_1.iloc[pf1].values,
↪   index=S1_subset_1.iloc[pf1].index.astype('int'))
peaks_S2 = pd.Series(S2_subset_1.iloc[pf2].values,
↪   index=S2_subset_1.iloc[pf2].index.astype('int'))
peaks_S3 = pd.Series(S3_subset_1.iloc[pf3].values,
↪   index=S3_subset_1.iloc[pf3].index.astype('int'))

# creates data frame with the peaks found in each sample (index is the
↪   wavenumber of the peaks)
peaks_frame = {'Peaks_S1': peaks_S1,
```

```
                 'Peaks_S2': peaks_S2,
                 'Peaks_S3': peaks_S3
                }


peaks_result = pd.DataFrame(peaks_frame)
peaks_stat = {'Mean': peaks_result.mean(axis=1, skipna=True),
              'Std Dev':peaks_result.std(axis=1, ddof=0, skipna=True)
             }
peaks_result = peaks_result.assign(**peaks_stat)
print(peaks_result)
```

Calculating area of the carbonyl region

For the calculation of the carbonyl index, the area of the carbonyl region (1675-1775 cm^{-1}) was calculated with numerical integration.

```
# CALCULATE AREA OF DEFINED REGION OF SPECTRA - in this case Carbonyl
↪  region for calculation of Carbonyl index (CI)


# Calculates area of subset (numerical integration of absorbance from
↪  1675-1775cm-1)
CI_S1 = np.trapz(S1_subset_1.values, x=S1_subset_1.index)
CI_S2 = np.trapz(S2_subset_1.values, x=S2_subset_1.index)
CI_S3 = np.trapz(S3_subset_1.values, x=S3_subset_1.index)


# Calculates the MEAN and STD DVE of the aread for the three samples
CI_0 = statistics.mean([CI_S1,CI_S2,CI_S3])
```

```
CI_0_SD = statistics.pstdev([CI_S1,CI_S2,CI_S3])


# Creates a dataframe with the results that can later be exported as .csv
↪  file
samples = pd.Series(['S1', 'S2', 'S3', 'Mean', 'STD DEV'])
CI = pd.Series([CI_S1, CI_S2, CI_S3, CI_0, CI_0_SD])
CI_frame = {'Sample': samples,
            'Carbonyl index': CI}
CI_result = pd.DataFrame(CI_frame)


print(CI_result)
```

Exporting the results

The results were exported as *comma separates values* (CSV) files that could be latter imported into graphing and data analysis software.

```
# EXPORTS RESULTS .CSV FILES


# Exports baseline corrected spectra as one .csv file
baseline_corrected_curves.to_csv('PEX-e2_T0-baseline-corrected-ALSS',
↪  sep=',', index=True, encoding='utf-8')  #  -----> modify file name
# Exports normalized spectra as one .csv file
normalized_curves.to_csv('PEX-e2_T0-normalized', sep=',', index=True,
↪  encoding='utf-8')  #  -----> modify file name
```

```
carbonyl_region.to_csv('PEX-e2_T0-carbonyl-region', sep=',', index=True,
↪  encoding='utf-8')  #  -----> modify file name
subset_2_region.to_csv('PEX-e2_T0-region-2', sep=',', index=True,
↪  encoding='utf-8')
subset_3_region.to_csv('PEX-e2_T0-region-3', sep=',', index=True,
↪  encoding='utf-8')

# Exports peaks detected
peaks_result.to_csv('PEX-e2_T0-carbonyl-peaks', sep=',', index=True,
↪  encoding='utf-8') #  -----> modify file name

# Exports results from area calculation
CI_result.to_csv('PEX-e2_T0-carbonyl-index', sep=',', index=True,
↪  encoding='utf-8')
```

References

(1) Hiles, M. Classifying Formulations and Tracking Accelerated Aging of Crosslinked Polyethylene Pipes by Applying Machine Learning Concepts to Infrared Spectra. Master Thesis, University of Guelph, Guelph, Canada, 2020.

(2) Eilers, P. H.; Boelens, H. F. *Baseline correction with asymmetric least squares smoothing*; Report 1.1, 2005; p 5.

(3) Futaana, Y.; Wieser, M.; Wieser, G. S. Python Library, Version 3.11.3. 2015; https://irfpy.irf.se/projects/ica/index.html, Copyright 2007-2015 Swedish Institute of Space Physics.
